# Unraveling the Physiological Mechanisms Underlying the Intracultivar Variability of Water Use Efficiency in *Vitis vinifera* “Grenache”

**DOI:** 10.3390/plants11213008

**Published:** 2022-11-07

**Authors:** Ignacio Buesa, Esther Hernández-Montes, Ignacio Tortosa, Gabriele Baraldi, Miquel Rosselló, Hipólito Medrano, Jose Mariano Escalona

**Affiliations:** 1 Research Group on Plant Biology Under Mediterranean Conditions, Department of Biology, University of Balearic Islands (UIB), 07122 Palma, Balearic Islands, Spain; 2 Plant Biology and Environment, Agro-Environmental and Water Economics Institute—University of Balearic Islands (INAGEA—UIB), 07122 Palma, Balearic Islands, Spain; 3 Scienze e Tecnologie Agro-Alimentari, Alma Mater Studiorum, Università di Bologna, 40127 Bologna, Italy

**Keywords:** carbon isotope discrimination, genotype evaluation, grape yield, growth rates, leaf gas exchange, leaf mass area, *Vitis vinifera*, water relations, water deficit

## Abstract

Selecting genotypes with a better capacity to respond and adapt to soil water deficits is essential to achieve the sustainability of grapevine cultivation in the context of increasing water scarcity. However, cultivar changes are very poorly accepted, and therefore it is particularly interesting to explore the intracultivar genetic diversity in water use efficiency (WUE). In previous studies, the cultivar “Grenache” has shown up to 30% variability in WUE. This research aimed to confirm the intracultivar variability and to elucidate the traits underlying this variability in the response to a water deficit by analyzing the growth rates, water relations, osmotic potential, leaf morphology, leaf gas exchange and carbon isotope discrimination in nine “Grenache” genotypes grown in pots during two seasons. The results showed lower differences in WUE and carbon isotope ratio than in previous field studies, but fairly good consistency in genotype ranking. Leaf mass area and osmotic potential did not underlie differences in stem water potential and in stomatal conductance. Overall, stomatal regulation and photosynthetic capacity seem to underlie differences in WUE among genotypes with an important environmental influence. These results confirm the ability to select clones with higher WUE and present an opportunity for the genetic improvement of WUE in grapevines.

## 1. Introduction

Adapting agriculture to climate change requires the efficient use of increasingly limited water resources [1,2]. Moreover, achieving this in an environmentally sustainable way is a major challenge [3]. In crops that are mainly rainfed, such as grapevine (*Vitis vinifera* L.), climate change poses a greater threat [3,4]. Potential adaptation measures include modifying training systems (e.g., goblet bush vines), trellised vineyards with greater spacing between rows or different row orientations, as well as the use of mulching [5,6,7].

Grapevine is a traditional and important crop in semi-arid regions because of its ability to adapt to limited water conditions [8,9]. This ability is linked to the regulation of water consumption by stomatal conductance regulation, among other hydraulic traits [10,11]. In grapevine, there is a vast genetic pool with large variability in drought stress responses among cultivars [12,13,14], but also among clones within the same cultivar [15,16]. This variability usually has resulted in differences of more than 30% in intrinsic water use efficiency (WUE_i_), both inter- and intracultivar. By definition, WUE_i_ is the ratio of CO_2_ assimilated per unit of water used. From a physiological perspective, it is the net photosynthesis (A_N_) divided by stomatal conductance (g_s_). To date, stomatal regulation and photosynthetic capacity have been shown to play a key role in WUE_i_ improvement among genotypes [17,18,19,20]. Nevertheless, both physiological mechanisms depend on many physiological traits, such as leaf morphology, osmotic adjustment, ABA dynamics, and aquaporins, among many others, which have been poorly explored in an integrated approach [11,21].

The intracultivar variability has been widely used in the wine industry, and the “Grenache” and “Tempanillo” cultivars are good examples of this exploitation. Both cultivars are of Spanish origin but are widely cultivated in wine-growing areas around the world [22,23]. In both cultivars, considerable intracultivar diversity has been reported, with 49 and 76 clones certified for “Tempranillo” and “Grenache”, respectively [24], including somatic mutations in both cultivars that have even led to white grape cultivars [25]. Intracultivar selection programs are currently an interesting tool for vineyard adaptation to climate change. Nurseries are already using WUE as a selection target in new clone breeding programs because this adaptation strategy is more widely accepted by winegrowers than cultivar changes, which are often limited by Protected Designations of Origin (DOP). Differences in stomatal behavior and leaf respiration rates have been reported between both cultivars [26,27,28]. Moreover, “Tempranillo” is an early ripening cultivar, while “Grenache” ripens late. This may be an important factor in the adaptation of viticulture to global warming in favor of late-ripening cultivars such as “Grenache” [29].

Buesa et al. [20] have quantified the intracultivar variability in WUE within “Grenache” and demonstrated its consistency over three seasons under field conditions. This ecophysiological evaluation was carried out through a multilevel methodology (leaf, grape and whole plant scale, i.e., vegetative development and yield), allowing the different genotypes to be ranked according to their WUE in an integrated way. However, the physiological underlying mechanism responsible for these differences remains largely unknown. Therefore, studies under controlled experimental conditions are needed to confirm these results, and especially to elucidate the factors underlying these differences in WUE [17]. In this sense, Tortosa et al. [19] have recently reported that physiological traits explained the differences in WUE between genotypes within the cultivar “Tempranillo”. Specifically, they linked differences in WUE mainly to mesophyll conductance and respiration rates, but also to maximal carboxylation and maximal electron transport rates. This knowledge is very important, as it would help us to understand the response strategies of genotypes to environmental factors.

Therefore, this work aimed to (1) confirm the intracultivar WUE variability within “Grenache” under highly controlled conditions of water availability (plants grown in pots), and (2) unravel the physiological traits underlying the responses to soil water deficits. This was achieved by monitoring the shoot growth rate (SGR), leaf area appearance rate (LAR), leaf mass area (LMA), stem water potential (Ψ_stem_), osmotic potential (Ψ_π_), and leaf gas exchange across a wide range of water statuses in nine “Grenache” genotypes during two seasons. In addition, at the end of the season, the vegetative biomass and grape yield were determined, as well as the carbon isotope ratio (δ^13^C), measured in grapes as a surrogate marker of WUE_i_.

## 2. Results

### 2.1. Meteorological Conditions

During the experimental seasons, from May to September, the average temperature and relative humidity were 24.8 °C and 72% and 23.5 °C and 64%, in 2020 and 2021, respectively (Supplementary Table S1). The accumulated reference evapotranspiration (ET_o_) and rainfall during these periods were 137 and 65 mm, and 131 and 25 mm, respectively.

### 2.2. Plant Water Relations and Net Photosynthesis Rates

**Figure 1:**
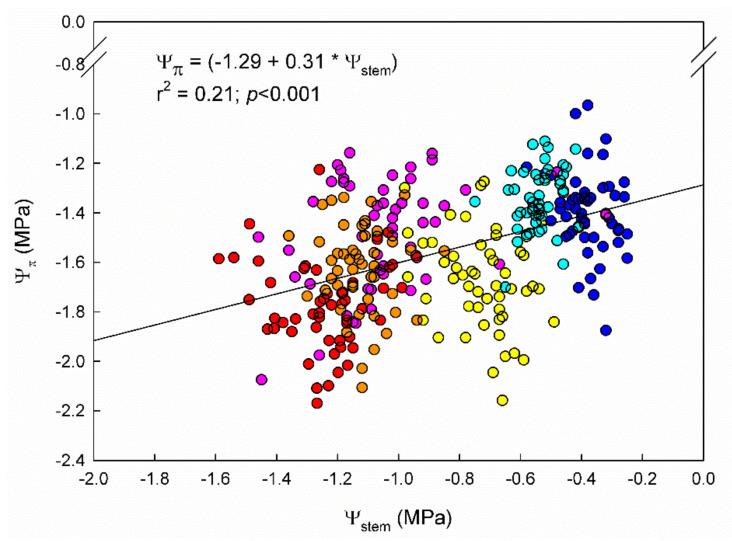
Fitted regression between stem water potential (Ψ_stem_) and leaf osmotic potential (Ψ_π_) during 2020 and 2021 seasons in 9 “Grenache” clones grown in Mallorca, Spain. Colored dots depict the day of the year (DOY) of measurement (cyan: 206; orange: 240; blue: 138; yellow: 152; pink: 179; and red: 211).

**Figure 2:**
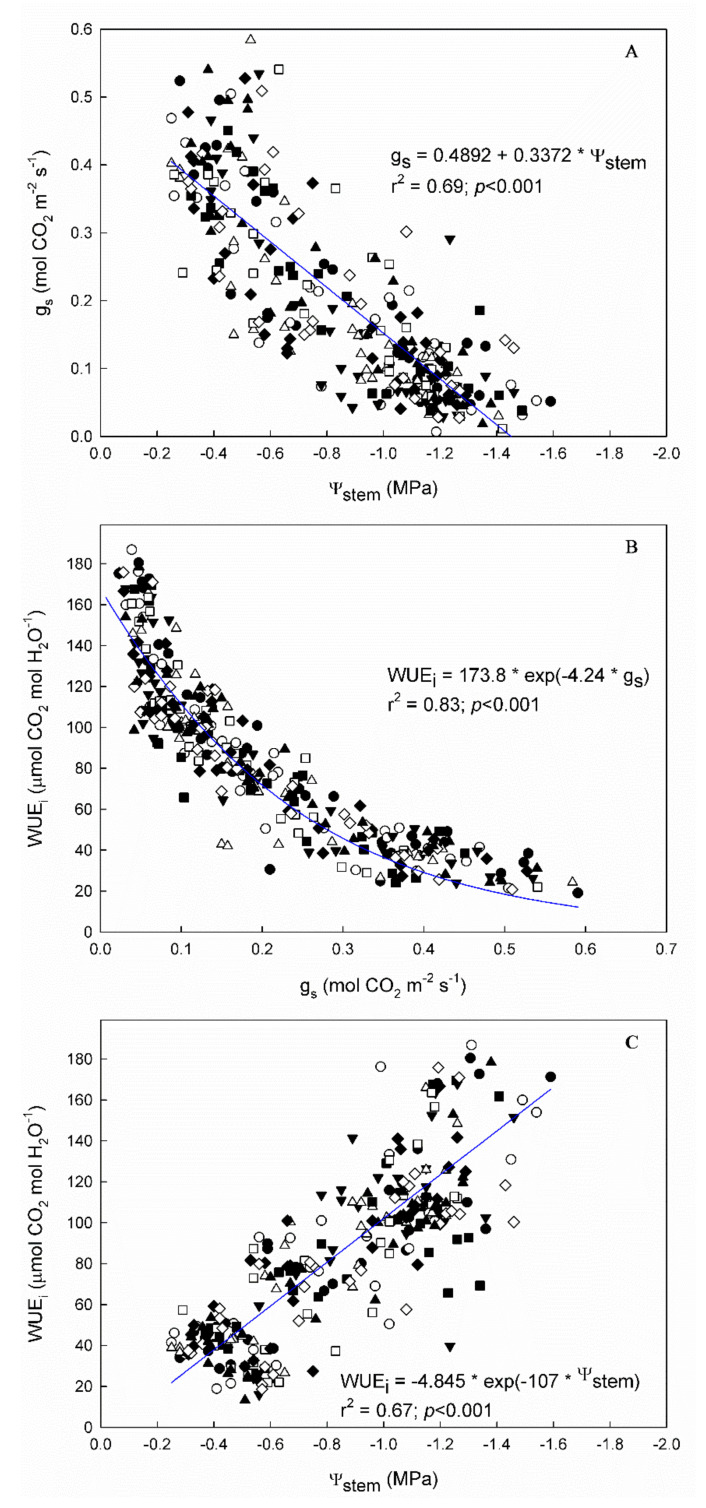
Linear relationships between (**A**) stomatal conductance (g_s_) and stem water potential (Ψ_stem_), (**B**) intrinsic water use efficiency (WUE_i_) and g_s,_ and (**C**) intrinsic water use efficiency (WUE_i_) and stem water potential (Ψ_stem_) in the 9 “Grenache” genotypes in the 2 experimental seasons (2020 and 2021), in Mallorca, Balearic Islands, Spain.

Vine water status, assessed by both stem water potential (Ψ_stem_) and leaf stomatal conductance (g_s_), showed a wide range of values across seasons in all “Grenache” genotypes (Figure 1 and Figure 2A). The Ψ_stem_ ranged from −0.25 to −1.60 MPa, and the g_s_ from 0.012 to 0.590 mol CO_2_ m^−2^ s^−1^. In all genotypes, the linear regression between both water indicators was highly significant (*p* < 0.001), but not very strong (r^2^ ranging from 0.61 to 0.80) (Supplementary Table S2).

On the one hand, significant differences in Ψ_stem_ among genotypes were observed, although only under WD conditions (Table 1). In 2020, noteworthy are the differences in Ψ_stem_ observed between EVENA-13 and ENTAV-136, which, on average, were up to 0.2 MPa. In 2021, the lowest Ψ_stem_ values were reached by ENTAV-136, differing from EVENA-11, 14, 15, and VNQ by more than 0.12 MPa. In both seasons, the other genotypes showed intermediate values between those of these genotypes, without being significantly different from them (Table 1).

Leaf osmotic potential (Ψ_π_), similarly to Ψ_stem_, showed a decreasing trend across the seasons (Table 1). Both parameters were significantly related, showing a mild, positive, linear relationship between them (Figure 1). The Ψ_π_ was, in all cases, lower than Ψ_stem_ (Table 1). Significant differences in Ψ_π_ among “Grenache” genotypes were found only in the 2020 season under WW conditions. At that time, EVENA-11 had a significantly more negative Ψ_π_ than EVENA-14, ENTAV-435, and VNQ.

On the other hand, g_s_ showed differences among genotypes in both WW and WD (Table 2). Under WW, EVENA-11 and RJ21 in 2020, and ARA-24 and EVENA-14 in 2021, were the only genotypes differing between them. Under WD, ENTAV-435 showed significantly lower g_s_ values than EVENA-15 and ENTAV-136 in 2020, while, in 2021, both ENTAV-435 and 136 showed lower values than VNQ (Table 2).

Regarding A_N_, significant differences among “Grenache” genotypes were found in 2020 under WD; in 2021, however, they were observed only under WW (Table 2). In 2020, ENTAV-435 and EVENA-13 had the lowest net photosynthesis rates, although only significantly lower compared to EVENA-15 and ENTAV-136. In 2021, ARA-24 showed a significantly higher A_N_ compared to EVENA-13 and 14, and RJ21.

Overall, the differences observed among genotypes in water relations and gas exchange parameters were not fully consistent across seasons.

### 2.3. Vine Growth

Vegetative development was assessed under well-watered (WW) and water deficit (WD) conditions by analyzing SGR, LAR, and LMA across both seasons (Table 3). Under WW, the SGR showed differences among genotypes only in 2021. In this season, the SGR values of EVENA-13 and VNQ were significantly lower than those of EVENA-11. Under WD, the SGR of EVENA-13 and EVENA-15 was significantly the lowest and the highest, respectively. Nevertheless, in 2021, there were no differences in SGR among genotypes under WD (Table 3). Regarding the LAR, under WW, there were no differences among genotypes in any season, in agreement with the similar Ψ_stem_ values observed (Table 1). Under WD, in 2020, the LAR of EVENA-15 was significantly higher than in EVENA-13 and 14, ENTAV-435, and RJ21, while, in 2021, EVENA-14 was the only one that showed an LAR higher than that of ARA-24. In general, both SGR and LAR showed negative correlations with Ψ_stem_, but in none of the parameters did the genotypes show a consistent pattern between seasons (Table 3).

The LMA showed differences among genotypes under WW but not under WD (Table 3). In both seasons, ARA-24 and EVENA-11 showed some of the highest LMA values, while VNQ was among the lowest. Nevertheless, there were genotypes that showed low LMA values in one season but high values in the other, such as EVENA-15 and EVENA-14.

### 2.4. Total Biomass

At the end of the experiment, leaf mass showed differences among “Grenache” genotypes only in 2021, where EVENA-14, ENTAV-435, and RJ21 were the ones that generated a greater leaf mass, while EVENA-15 had the lowest (Table 4). Differences among genotypes in leaf mass were up to 44%. Moreover, shoot mass was also only affected in 2021. It is noteworthy that ARA-24 accumulated less mass in the shoots than most of the other genotypes. Regarding grape yield, ARA-24 and ENTAV-136 yielded 57% more than EVENA-14 and 15, while the other genotypes showed intermediate values, without differing from the others. Total biomass was not affected by genotype in 2020, whereas, in 2021, ARA-24 showed the lowest values, while EVENA-14 and ENTAV-435 showed significantly the highest (Table 4).

### 2.5. Water Use Efficiency

Water use efficiency results at the leaf level (WUE_i_) are split by year and watering condition (Table 5). Under WW, there were differences in WUE_i_ among genotypes only in 2020. At that time, EVENA-11 showed higher values than RJ21 and VNQ. Remarkably, under WD, the relative differences in WUE_i_ among genotypes were fairly consistent between seasons. EVENA-13 was the “Grenache” genotype with the lowest WUE_i_ in both seasons, followed by VNQ, while EVENA-14 and ENTAV-136 showed the highest values in 2020 and 2021, respectively (Table 5).

**Figure 3:**
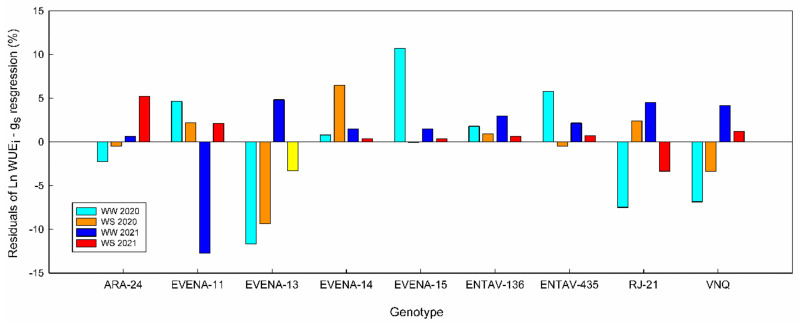
Residuals of the water use efficiency over stomatal conductance (WUE_i_–g_s_) linearized regression for each “Grenache” genotype. Well-watered 2020 (cyan); water stress 2020 (orange); well-watered 2021 (blue); water stress 2021 (red).

As expected, the WUE_i_ of the genotypes decreased exponentially with g_s_ and linearly with Ψ_stem_ (Figure 2B,C). The Ln WUE_i_–g_s_ calculated for each genotype showed r^2^ values higher than 0.75 in all cases (Supplementary Table S3). Differences in the slope of these regressions were found only between the ones of ARA-24 and EVENA-13. Despite the fact that the r^2^ value of the general WUE_i_–g_s_ relationship was 0.82, and therefore also quite strong (Figure 2B), the residuals of some genotypes were greater than 10% (Figure 3). In Figure 3, we show the residuals of the linearized relationship between WUE_i_ and g_s_ in the nine “Grenache” genotypes, for each date of measurement across the two experimental seasons—that is, the deviation of each genotype with respect to the general “Grenache” behavior. The analysis of residuals in the ln WUE_i_–g_s_ regressions for each date of measurement indicates that there were differences in WUE_i_ among genotypes for similar g_s_ rates. Although there was great variability in the residuals among dates, it is noteworthy that EVENA-13, followed by RJ21 and VNQ, was the genotype that showed the lowest WUE_i_ compared to the others. On the contrary, ARA-24, EVENA-11 and ENTAV-136 and 435 showed positive residuals on most of the dates, especially when the vines were subjected to WD (Figure 3).

**Figure 4:**
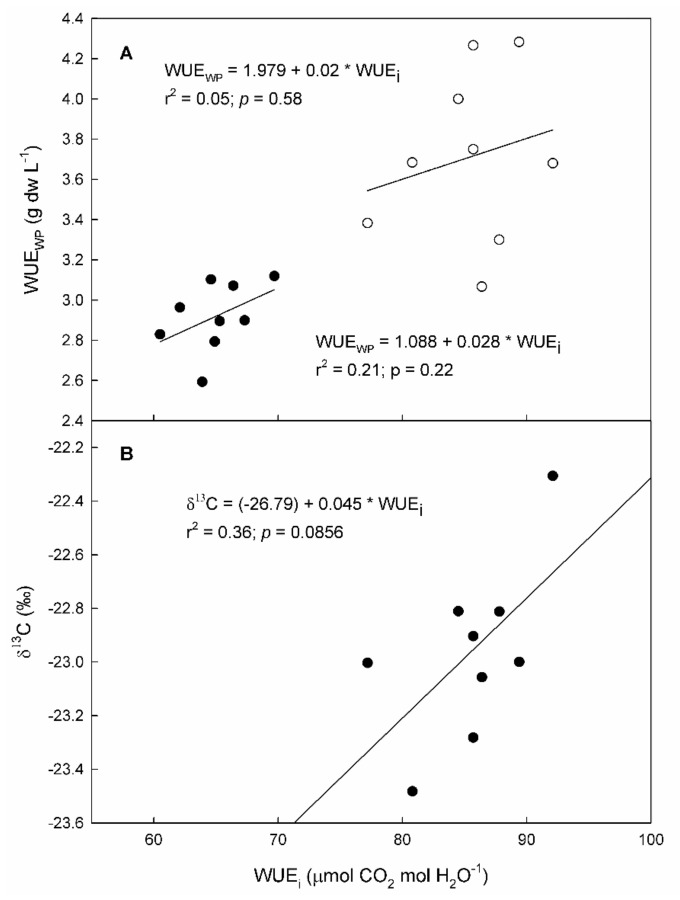
Relationship between intrinsic water use efficiency (WUE_i_) and (**A**) whole plant water use efficiency (WUE_WP_) and (**B**) carbon isotope ratio (δ^13^C) measured on 9 “Grenache” genotypes in 2020 (black dots) and 2021 (white dots).

Whole plant water use efficiency (WUE_WP_) differed among genotypes by 17 and 28% in the 2020 and 2021 seasons, respectively (Table 5). In 2020, ENTAV-136 showed significantly lower values than EVENA-11, ENTAV-435, and RJ21. In 2021, ARA-24 was the one that was highlighted for its lowest WUE_WP_, while EVENA-14, ENTAV-435, and RJ21 showed significantly the highest. The WUE_WP_ was not significantly related to WUE_i_, although a positive trend between both WUE levels was observed in both seasons (Figure 4).

### 2.6. Carbon Isotope Ratio

Genotypes showed differences in carbon discrimination in grapes in response to water deficits (Table 5). Differences in δ^13^C among genotypes reached up to 1.2‰. Genotype ENTAV-136 showed the least negative values, differing significantly from ARA-24, EVENA-14, and VNQ. The other genotypes did not show significant differences in this parameter with respect to any genotype. Moreover, the δ^13^C in grapes showed a positive tendency (*p*-value = 0.086) to be related to WUE_i_ (Figure 4B).

### 2.7. Ranking Genotypes in WUE

The ranking of “Grenache” genotypes showed a marked seasonal effect both in WUE_WP_ and WUE_i_ (Table 6). Classifying genotypes based on WUE_WP_ showed higher interannual variability than WUE_i_. However, in neither of the two WUE indicators were the results completely opposite between years for any genotype. The relative position of the genotypes was fairly similar among all three WUE indicators, with the exception of ARA-24.

Within each level, some genotypes stood out as more or less efficient (Table 6). This was the case for EVENA-11 and 14, ENTAV-136 and 435, and RJ21, which were found to be the most efficient. In contrast, EVENA-13 and 15 and VNQ were ranked as the least efficient.

## 3. Discussion

The genetic variability in WUE within the “Grenache” cultivar was confirmed under pot conditions at the leaf, grape, and whole plant levels. This agrees with the previous ecophysiological evaluation of “Grenache” genotypes carried out under field conditions during three seasons [20]. Nevertheless, the stability in genotype classification did not fully match. There were some genotypes that showed consistently better WUE in both environments, such as EVENA-14, ENTAV-136 and 435, or worse, such as EVENA-13 and 15 (Table 6). However, other genotypes showed differences or even a contrasting response to the environment, such as RJ21. This could be due to the fact that differences in WUE among genotypes depend on the range of water status [17,20]. In an attempt to unravel the physiological mechanisms underlying genotype responses in WUE to water deficits, greater control of soil water availability (pots) and more physiological measurements (SGR, LAR, Ψ_π_, evaluation of total biomass, etc.) were carried out. In Buesa et al. [20], differences between genotypes were found to be consistent between seasons and were greater than 30% in WUE_i_, 61% in yield and around 10% in δ^13^C. However, in the current study, under more controlled conditions in pots, the differences in the indicators of water use efficiency were milder. Specifically, they were 15% in WUE_i_, 57% in yield, 23% in total biomass and 5% in δ^13^C (Table 4 and Table 5). However, the range of water statuses to which the vines were subjected was comparable between experiments (Figure 2 and [20]). Specifically, g_s_ was higher than 0.150, between 0.150 and 0.075 and lower than 0.075 mol H_2_Om^−2^s^−1^ under non-, moderate and severe water stress conditions, respectively [30].

The question that arises is whether the lower differentiation of genotypes in pots is due to the greater control of environmental conditions, i.e., soil water availability, or because the response is different depending on the environment. Our hypothesis is a combination of the two, but with greater importance given to the second explanation. On the one hand, it is plausible that, in the field, there is more variation in soil water availability than in pot conditions. Therefore, the acclimatization processes during the soil water deficit could have been somewhat different among genotypes across the field experiment [31]. However, this effect is considered minor given the homogeneity of the plot and experimental design used [20]. On the other hand, the ecophysiological evaluation methodology implemented to assess water use efficiency among genotypes was carried out in multiple levels (leaf, grape, and plant level). In this way, the variation in the response of genotypes can be characterized under water stress conditions by three complementary approaches (WUE_i_, δ^13^C, and biomass production). The robustness of the statistical analysis used meant that the g_s_ data made it possible to evaluate each plant as a function of vine water status, using stomatal conductance as a reference [10]. Using water status as a reference, i.e., g_s_, reinforces the hypothesis that the response of “Grenache” genotypes varies according to the environment. Nonetheless, environments with a high vapor pressure deficit led to higher stomatal regulation, and a value below 0.1 mol CO_2_ m^−2^ s^−1^ induces higher variability in WUE_i_ (Figure 2B). This could be the case when comparing genotypes in such contrasting environments as Mallorca and Navarra.

Differences among genotypes observed in pots showed lower consistency across the experiment than in the field [20]. This was despite the meteorological conditions being fairly similar between the two experimental seasons (Supplementary Table S1). The greater differences observed in pots than in the field are in agreement with a previous evaluation of “Tempranillo” genotypes under both pot and field conditions [17]. Regarding the consistency between field and pot condition results, relative differences between genotypes were not fully consistent, but there was good overall agreement (Table 6). In our experiment, the most efficient genotypes at the leaf level were EVENA-14 in 2020 and ENTAV-136 in 2021, and EVENA-13 was the least efficient in both seasons (Table 2). In Buesa et al. [20], regardless of seasonal variability, the former were ranked as the second and fourth best genotypes of 13 genotypes, while EVENA-13 was again among the least efficient ones. In terms of productivity, in both experiments, ENTAV-136 and EVENA-14 were among the most and least productive, respectively, unlike ARA-24 (Table 5). Regarding the surrogate indicator of WUE, i.e., δ^13^C, ENTAV-136, and ARA-24 showed good agreement in both experiments, but not EVENA-14 and VNQ (Table 5). Given the degree of general consistency at the different WUE levels, it can be confirmed that the differences have a genetic origin. Notwithstanding, the environment has also been a relevant factor. These results are in agreement with those observed in “Tempranillo”, in which, despite the inter-seasonal variability, consistency in the WUE response of genotypes to water deficit has also been detected across environments [17,18,19].

Under potted conditions, the vine water status was remarkably affected by the “Grenache” genotypes when assessed by gas exchange, i.e., g_s_ (Table 2); however, differences in Ψ_stem_ among genotypes were observed only under WS conditions (Table 1 and Table 2). This discrepancy suggests differences in stomatal control among clones, i.e., g_s_–Ψ_stem_ relationship. Since the range of g_s_ in our experiment was wide, it enabled us to compare the slopes of the WUE_i_–Ψ_stem_ regressions among genotypes. In fact, the g_s_–Ψ_stem_ regression of RJ21 showed significant differences in its slope compared to those of ENTAV-435 and VNQ (Supplementary Table S2), confirming genetic differences in stomatal regulation. In any case, the stomatal behavior was isohydric in all the “Grenache” genotypes, as the slope of the g_s_–Ψ_stem_ regressions suggests (Figure 2A). Bota et al. [13], in a work assessing the differences among grapevine cultivars in their stomatal behavior and WUE under progressive water stress, stated that slopes higher than 0.25 would indicate tight stomatal regulation. This was our case, with slopes ranging from 0.30 to 0.40, which is consistent with previous works that classified “Grenache” as a strong water-saving grapevine cultivar [12,14,26,27].

Comparing the individual WUE_i_–g_s_ regressions for each genotype with the ones reported by Buesa et al. [20], differences between them stand out (Supplementary Table S3). In our study, these slopes ranged from −3.22 to −4.14, while, under field conditions, they ranged from −2.13 to −2.79. These differences confirm the environmental effect in the WUE responses of the “Grenache” genotypes and imply that, for a given g_s_, the genotypes grown under pots had a systematically higher A_N_. This is the opposite of what was observed in “Tempranillo” grown in pots and in the field by Tortosa et al. [17]. The plant’s nutritional status could be underlying these responses, but this is unlikely in the case of “Grenache” because both experiments were fertigated. Thus, the more reactive relationship of WUE_i_ to g_s_ under pot than field conditions suggests physiological shifts in the acclimation to water stress. In this sense, plant water relations play a key role, including leaf osmotic potential and leaf hydraulic conductance [11,20]. Water potential depends on a number of factors that were different between the field and pot experiments, such as the evaporative demand, the hydraulic architecture of the plant, and the soil texture and depth [32]. In this regard, a very important factor in the response of genotypes to water deficit is the rootstock [33]. In the field experiment, the “Grenache” genotypes were grafted onto the 110-Richter rootstock, while, in pots, they were ungrafted. It is well known that rootstocks can influence grapevine responses to drought through their influence on the vigor and productivity of scions, namely by affecting hydraulic traits, water uptake/transport capacity, osmotic adjustment, and leaf gas exchange [34,35,36].

In our trial, the observed differences among genotypes in Ψ_stem_ were not caused by differences in Ψ_π_ (Table 1), although, as expected, both followed a steady decrease across the season (Figure 1) [37]. This suggests that the slight differences observed in Ψ_stem_ among genotypes under WD (Table 1) were not due to differences in osmotic adjustment to water stress [36]. Therefore, it might have been due to the tension generated by the leaf transpiration at the whole vine level. In fact, Dayer et al. [14] observed that the capacity of genotypes to increase water use under well-watered conditions was strongly associated with hydraulic traits. However, Tortosa et al. [19] did not report differences in leaf hydraulic traits (osmotic potential at turgor loss point, cell wall elasticity, or cell capacitance) that would explain differences in gas exchange parameters (g_s_ and WUE_i_) among “Tempranillo” genotypes.

As expected, vines invested in LMA throughout its development in response to climate conditions (Table 3). This is due to the thickening of the cell wall as a function of both water stress and leaf age. For this reason, differences in LMA were expected mainly under WS, but the opposite was found (Table 3). Roig-Oliver et al. [31] showed also in “Grenache” cultivars that modifications in the cell wall due to environmental acclimation can play a significant role in leaf physiology, i.e., A_N_ and water relations. In our case, since our genotypes showed no differences among them in LMA under WS conditions, the physiological differences observed among them are ruled out as being generated by differences in leaf mass area. On the other hand, differences in LMA between potted and field grown vines may be responsible for the environmental effect in genotypes” responses to water deficits. Under field conditions, plants were grown under a progressive drought throughout the season, while, in potted plants, water stress occurred earlier in the season. This means that the potted vines could have had a higher LMA because the leaves were exposed to a longer water stress period [13]. A higher LMA induces thicker and/or denser leaves, and consequently more photosynthetic tissue per area unit [38]. Other factors that can explain these differences could be the nitrogen content in the leaf, or higher mesophyll conductance [39]. Variability in mesophyll conductance was indeed associated with grapevine WUE_i_ [40] and has recently been reported to be an important trait of differentiation within “Tempranillo” genotypes [19]. Nevertheless, mesophyll conductance in grapevines could not be explained by anatomical variability, suggesting that biochemical mechanisms play an important role in WUE_i_ [40].

In our experiment, WUE_i_ was not significantly correlated to WUE_WP_ (Figure 4). In the grapevine, it is recognized that WUE_WP_ can be decoupled from WUE_i_ [19,41,42]. Several factors were proposed to explain these discrepancies, including canopy light interception, root respiration, leaf respiration, and transpiration at night [43]. Nonetheless, the low SGR and LAR observed in EVENA-13, RJ21 and VNQ agreed with their negative residuals in the WUE_i_–g_s_ regression (Table 3 and Figure 3). The analysis of residuals, however, only confirms the high SGR and LAR for EVENA-11. Moreover, the efficiency in carbon assimilation is not only related to yield, but also to the total biomass. This proves the importance of carbon partitioning on WUE in terms of yield (Table 4). In this regard, the genotypes showed important differences among them. For instance, ARA-24 was the most productive, but the one that invested the least in vegetative biomass, and the opposite occurred with EVENA-14. This makes the study of WUE much more complex. For this reason, a multilevel approach is needed to obtain robust conclusions.

In this sense, δ^13^C is very useful, as it integrates the entire grape ripening period [44]. The δ^13^C values observed in our trial were overall within the expected range for “Grenache” berries at harvest (Table 5) [44,45] and confirmed that the vines had suffered severe water stress [20]. Genotypes such as ARA-24, EVENA-14, and VNQ showed δ^13^C values among the most negative genotypes (Table 5), which are indicative of lower WUE_i_ [45]. Meanwhile, ENTAV-136 showed the least negative values, and hence higher WUE_i_ than the others [13,46], which was in agreement with what was observed at the leaf level, except for EVENA-14 (Figure 4). VNQ showed the most negative δ^13^C value, in agreement with its low WUE_i_ (Table 2), which is also related to lower water stress (Table 1).

## 4. Materials and Methods

### 4.1. Site Description and Plant Material

The experiment was conducted outdoors for two consecutive seasons (2020–2021) in the experimental field of the University of Balearic Islands (UIB) (39°38′15″ N 2°38′51″ E). Two-year-old ungrafted plants were transplanted during winter into pots after trimming the root tips. The pots were 20 L in volume and were filled with a mixture of organic substrate (blond peat) and perlite (4:1). A 2–3 cm layer of perlite was placed on top of the substrate to minimize soil water evaporation. Each vine was irrigated through two irrigation micro-tubes with pressure-compensated drippers of 0.5 L h^−1^. Irrigation was applied during the whole experiment, including NPK and microelement nutrient solution to maintain the plants at an optimum nutrient status.

The climate of the area was classified as Mediterranean and semi-arid. Meteorological data were recorded by an automatized meteorological station located in the UIB’s experimental farm (Meteodata 3000C, Geonica S.A., Madrid, Spain).

The plant material used was 9 genotypes of the red “Grenache” cultivar (*Vitis vinifera* L.): ARA-24, EVENA-11, EVENA-13, EVENA-14, EVENA-15, ENTAV-136, ENTAV-435, RJ21, and VNQ (Vitis Navarra S.L., Larraga, Navarra, Spain). Vines were pruned to a 2-bud count per vine and trained vertically with two canes. Canopy management included green pruning before bloom and no shoot trimming, while all secondary shoots were removed weekly. This was completed to facilitate the determination of vegetative growth rates and to reduce leaf self-shading.

### 4.2. Experimental Design

The experimental design consisted of nine “Grenache” genotypes arranged in 6 complete blocks with one biological replicate per block (*n* = 6), for a total of 54 experimental plants. The plants were irrigated to field capacity twice a day, in order to meet their evapotranspiration demand until shoots reached a 1.5 m height. Once the experiment began, plants were maintained under field capacity for 20 days approximately (well-watered; WW). Afterward, a soil water deficit (WD) was imposed to induce progressive water stress in the vines, first by establishing a mild, moderate and finally, severe soil water deficit [30], corresponding to g_s_ values of 0.200–0.100, 0.150–0.075, and lower than 0.075 mol H_2_Om^−2^s^−1^, respectively. Each condition was maintained for at least 15 days.

For each season and date of measurement, all physiological determinations were carried out in each biological replicate (*n* = 6)—first, under WW conditions, and, after a minimum of two weeks of WD, the following set of physiological determinations. In 2020, these determinations were performed at two points in time, namely 24 July (WW) and 27 August (WD), while in 2021, it was performed at four points in time: 15 May (WW), 1 June (WD1), 28 June (WD2) and 30 July (WD3). In addition, at the end of the experiment, vegetative biomass and grape yield were determined in each biological replicate (*n* = 6).

### 4.3. Vegetative Growth

The shoot growth rate (SGR) was calculated by the average difference in shoot length of the two main shoots per plant between dates. The leaf area appearance rate (LAR) was the average difference in leaf number in the two shoots per plant between dates. In 2020, SGR and LMA were determined in each biological replicate on 3 occasions under WW (24 and 31 July, and 7 August), and 3 under WD (18 and 27 August, and 1 September). In 2021, these parameters were determined on 3 occasions under WW (6, 13 and 19 May) and 5 occasions under WD (28 May, 2, 9, and 24 June, 7 July and 9 August).

The leaf mass area (LMA) was calculated in each biological replicate using three circular samples of 2 cm diameter, taken with a punch from leaves similar to those used for physiological determinations and on the same dates. These samples were oven dried at 70 °C for 72 h before weighing.

Vegetative biomass was obtained separately for leaves and shoots in each experimental vine. Three subsamples of both tissues were taken at the end of the season of approximately 50 g of leaves and 100 g of shoots per genotype for oven drying. The difference between fresh and dry mass was used to estimate the total dry mass of each biological replicate (*n* = 6). In addition, in 2021, the grape yield was harvested and weighed in each experimental vine (*n* = 6). Previously, at the bloom stage, the crop load was adjusted to 2 clusters in each vine. The total biomass was estimated as the sum of the dry weight of leaves and shoots in 2020, while in 2021, the grape yield was also included. Whole plant WUE (WUE_WP_) was calculated as the ratio of total biomass to irrigation water applied during the deficit irrigation period.

### 4.4. Water Relations

Grapevine water status was determined by midday stem potential (Ψ_stem_), measured with a Scholander pressure chamber (M 1505D, MMM Tech Support, Berlin, Germany). One fully expanded leaf per plant was covered with an opaque zip envelope for at least an hour prior to its measurement at solar noon (13:00–15:00).

In addition, leaf osmotic potential (Ψ_π_) was determined as one of the most important components of the plant’s water potential. This determination was performed in samples of the same leaves in which Ψ_stem_ was measured. Each leaf was first frozen and stored at −20 °C and finally measured with a digital osmometer (Vapor Pressure Osmometer, ELITechGroup, Model 5600, Puteaux, France).

### 4.5. Leaf Gas Exchange

The stomatal conductance (g_s_) and net photosynthesis (A_N_) were measured in one leaf per plant using an infrared gas exchange analyzer (Li-6400xt, Li-cor Inc., Lincoln, NE, USA). The CO_2_ concentration inside the chamber was 400 μmol CO_2_ mol^−1^ air. The chamber used had an area of 6 cm^2^ exposed to environmental light radiation, with photosynthetic active radiation (PAR) always above 1200 μmol m^−2^ s^−1^. All measurements were performed between 11:30 and 13:00 solar time on the same dates as Ψ_stem_ determination. Intrinsic water use efficiency (WUE_i_) was calculated as the A_N_ to g_s_ ratio.

### 4.6. Carbon Isotope Ratios

The carbon isotope ratio (δ^13^C) was determined from the samples of 20 berries per plant used for berry mass determination in 2021. First, seeds were removed and then oven-dried at 85 °C for 10 days. Dried berries were ground at 25 Hz until powdered (Mixer Mill MM 200, Retsch, Düsseldorf, Germany). This powder was enclosed in zinc capsules of 2 ± 0.05 mg and then injected into a continuous-flow isotope ratio mass spectrometer (Thermo Finnigan MAT DELTA^plus^ XP, Barkhausenstr, Bremen, Germany). Peach leaf (NIST 1547) standards were run every eight samples [47]. The carbon isotope ratio (δ^13^C) was calculated as δ^13^C (‰) = (R_sample_/R_standard_ − 1) × 1000, where R_sample_/R_standard_ referred to a Pee Dee Belemnite standard.

### 4.7. Ranking Genotypes in WUE

Genotypes were classified by WUE and evaluated at three levels: at the whole plant level by WUE_WP_, at the leaf level by WUE_i_, and at the grape level by δ^13^C. The ranking was established in three categories, where 1, 2, and 3 denote high, medium, and low WUE according to the significant differences in each of the three levels of evaluation. For a more integrative comparison, the ranking values for each of the three WUE levels were averaged to rank each genotype with a single value.

### 4.8. Statistical Analysis

Data were checked for normality, and when datasets were not normal, i.e., WUE_i_, a logarithmic transformation was used. The evaluation of the effects of the genotype (G), date (D), and their interactions (GxD) on the studied variables was carried out by means of a two-way analysis of variance (ANOVA). Within each season, as the GxD had no significant effect on any of the measured variables, the 2021 data under water deficit are shown averaged over the three dates. A one-way ANOVA was used to evaluate the effects of the genotypes on all the variables. Mean separation was assessed with the Duncan post hoc test. Moreover, the Ln WUE_i_–g_s_ relationship was used to assess differences between genotypes by analyzing its residuals with respect to the general regression. In addition, the WUE_i_–g_s_ regressions obtained specifically for each genotype across seasons were compared based on differences in their slopes by a two-way analysis of covariance (ANCOVA) (see Tortosa et al. [18] and Buesa et al. [20]). All analyses were performed with the Statgraphics Centurion XVI package (version 16.0.07) (Statgraphics Technologies, The Plains, VA, USA). Differences were accepted with *p*-value < 0.05. Regressions were obtained using SigmaPlot (version 11.0) (Systat Software, San Jose, CA, USA).

## 5. Conclusions

The intracultivar variability in WUE within the “Grenache” cultivar was confirmed, with relative consistency at the leaf (A_N_/g_s_), grape (δ^13^C) and whole plant levels. However, large seasonal variability was found in genotype responses, with some having more stable responses than others. Compared to previous studies under field conditions, the differences in WUE among “Grenache” genotypes were half as great as under the more controlled pot conditions. Nevertheless, there was good consistency in the ranking of genotypes between experiments, confirming the genetic variability. Notwithstanding, the intracultivar variability in WUE of the “Grenache” cultivar appears to be environmentally dependent. Analyses of leaf mass area and osmotic potential did not provide insights into the physiological processes underlying the differences in WUE. Stomatal regulation and photoassimilate partitioning seem to be the physiological processes that govern the intracultivar variability in “Grenache” performance under water stress conditions. Further studies focusing on hydraulic traits, primary and secondary metabolism, and hormonal signals may help to explain these differences in WUE.

## Figures and Tables

**Table 1:** Stem water potential (Ψ_stem_) and osmotic potential (Ψ_π_) for each of the 9 “Grenache” genotypes under well-watered (WW) and water deficit (WD) conditions, grown in Mallorca, Balearic Islands, Spain, during 2020 and 2021 seasons.

Genotype	Ψ_stem_ (MPa)						Ψ_π_ (MPa)				
	2020			2021			2020			2021	
	WW	WD		WW	WD		WW		WD	WW	WD
ARA-24	−0.52	−1.13	abc	−0.38	−1.05	ab	−1.33	abc	−1.65	−1.39	−1.65
EVENA-11	−0.55	−1.10	abc	−0.37	−0.94	b	−1.47	a	−1.68	−1.46	−1.61
EVENA-13	−0.58	−1.23	a	−0.40	−1.01	ab	−1.43	ab	−1.79	−1.30	−1.64
EVENA-14	−0.55	−1.11	abc	−0.39	−0.99	b	−1.24	c	−1.61	−1.28	−1.61
EVENA-15	−0.57	−1.10	bc	−0.36	−0.96	b	−1.37	abc	−1.61	−1.40	−1.71
ENTAV-136	−0.51	−1.03	c	−0.34	−1.11	a	−1.33	abc	−1.54	−1.47	−1.63
ENTAV-435	−0.53	−1.19	ab	−0.43	−1.00	ab	−1.30	bc	−1.60	−1.49	−1.63
RJ21	−0.58	−1.18	ab	−0.38	−1.04	ab	−1.43	ab	−1.65	−1.40	−1.65
VNQ	−0.49	−1.12	abc	−0.40	−0.97	b	−1.23	c	−1.61	−1.39	−1.63

Within each row, different letters indicate significantly different results at *p* < 0.05 (Duncan test).

**Table 2:** Leaf stomatal conductance (g_s_) and net photosynthesis (A_N_) for each of the 9 “Grenache” genotypes under well-watered (WW) and water deficit (WD) conditions, grown in Mallorca, Balearic Islands, Spain, during 2020 and 2021 seasons.

Genotype	g_s_ (mol CO_2_ m^−2^ s^−1^)								A_N_ (µmol CO_2_ m^−2^ s^−1^)					
	2020				2021				2020			2021		
	WW		WD		WW		WD		WW	WD		WW		WD
ARA-24	0.400	ab	0.094	ab	0.428	b	0.131	ab	10.9	9.5	ab	17.9	d	12.9
EVENA-11	0.291	a	0.103	ab	0.387	ab	0.119	ab	9.2	10.7	ab	15.3	bcd	11.8
EVENA-13	0.372	ab	0.079	ab	0.341	ab	0.136	ab	9.8	7.6	a	14.5	ab	11.3
EVENA-14	0.482	ab	0.101	ab	0.334	a	0.131	ab	12.1	10.8	ab	12.3	a	11.3
EVENA-15	0.439	ab	0.123	b	0.356	ab	0.118	ab	12.9	12.0	b	16.1	bcd	11.4
ENTAV-136	0.459	ab	0.127	b	0.389	ab	0.103	a	12.0	12.1	b	16.7	bcd	10.0
ENTAV-435	0.496	ab	0.070	a	0.377	ab	0.098	a	12.6	7.4	a	17.2	cd	9.5
RJ21	0.546	b	0.092	ab	0.339	ab	0.125	ab	12.3	9.6	ab	15.1	bc	11.2
VNQ	0.520	ab	0.080	ab	0.382	ab	0.151	b	11.7	8.1	ab	16.2	bcd	12.8

Within each row, different letters indicate significantly different results at *p* < 0.05 (Duncan test).

**Table 3:** Shoot growth rate (SGR), leaf area appearance rate (LAR) and leaf mass area (LMA) for each of the 9 “Grenache” genotypes under well-watered (WW) and water deficit (WD) conditions during 2020 and 2021 seasons.

Genotype	SGR (cm day^−1^)						LAR (n day^−1^)						LMA (g m^−2^)					
	2020			2021			2020			2021			2020			2021		
	WW	WD		WW		WD	WW	WD		WW	WD		WW		WD	WW		WD
ARA-24	1.7	1.9	bc	3.0	ab	1.9	0.7	0.44	ab	0.42	0.19	a	79.8	b	75.4	62.4	b	75.4
EVENA-11	2.4	1.7	bc	3.3	b	2.3	0.64	0.40	ab	0.43	0.22	ab	80.8	b	77.5	63.0	b	77.2
EVENA-13	1.5	0.9	a	2.7	a	2.2	0.67	0.31	a	0.40	0.22	ab	70.5	ab	76.6	60.2	ab	76.8
EVENA-14	2.1	1.2	ab	3.0	ab	2.3	0.73	0.31	a	0.45	0.24	b	76.6	ab	73.9	54.4	a	70.9
EVENA-15	2.1	2.0	c	2.9	ab	2.0	0.73	0.45	b	0.43	0.21	ab	67.1	a	75.5	65.3	b	78.4
ENTAV-136	1.8	1.7	bc	3.1	ab	2.1	0.63	0.42	ab	0.40	0.20	a	77.9	ab	78.0	67.2	b	75.3
ENTAV-435	2.3	1.2	ab	3.1	ab	2.2	0.7	0.30	a	0.40	0.21	ab	75.3	ab	76.4	64.0	b	76.4
RJ21	2.2	1.5	abc	3.0	ab	2.3	0.79	0.30	a	0.44	0.21	ab	76.9	ab	78.4	59.6	ab	75.6
VNQ	2.0	1.5	abc	2.7	a	2.2	0.74	0.38	ab	0.40	0.22	ab	67.3	a	81.4	59.6	ab	74.3

Within each row, different letters indicate significantly different results at *p* < 0.05 (Duncan test).

**Table 4:** Grapevine biomass for each of the 9 “Grenache” genotypes at the end of the experiment in 2020 and 2021, in Mallorca, Spain.

Genotype	Leaf Mass			Shoot Mass			Yield		Total Biomass (g dw)		
	(g dw)			(g dw)			(g fw)				
	2020	2021		2020	2021		2021		2020	2021	
ARA-24	71.4	172.4	b	44.4	131.4	a	428.8	b	115.8	304	a
EVENA-11	75.8	198.8	b	49.0	173.7	b	302.8	ab	124.8	372.3	bc
EVENA-13	69.9	178.2	ab	43.2	157.3	ab	260.5	ab	124.2	335.7	ab
EVENA-14	70.0	247.7	c	46.0	174.1	b	173.5	a	113.8	421.7	d
EVENA-15	69.2	139.1	a	42.5	188.8	b	181.2	a	113.1	327.8	ab
ENTAV-136	60.8	188.4	ab	42.9	175.4	b	402.8	b	116.3	364.0	bc
ENTAV-435	72.6	237.5	c	50.2	185.9	b	330.2	ab	120.2	423.3	d
RJ21	72.5	233.1	c	51.6	161.4	ab	352.0	ab	119.4	394.6	cd
VNQ	74.5	195.7	ab	44.9	170.0	b	325.8	ab	107.8	365.8	bc

Within each row, different letters indicate significantly different results at *p* < 0.05 (Duncan test).

**Table 5:** Intrinsic water use efficiency (WUE_i_), whole plant water use efficiency (WUE_WP_), and carbon isotope ratio in grapes (δ^13^C) for each of the 9 “Grenache” genotypes under well-watered (WW) and water deficit (WD) conditions during 2020 and 2021 seasons in Mallorca, Spain.

Genotype	WUE_i_ (µmol CO_2_ mol H_2_O^−1^)							WUE_WP_ (g L^−1^)				δ^13^C (‰)	
	2020				2021			2020		2021		2021	
	WW		WD		WW	WD							
ARA-24	28.4	ab	102.3	ab	42.1	115.6	ab	2.9	ab	3.1	a	−23.1	a
EVENA-11	35.7	b	103.8	ab	39.7	117.4	ab	3.1	b	3.8	bc	−22.9	ab
EVENA-13	26.4	ab	94.7	a	42.7	102.3	a	2.8	ab	3.4	ab	−23.0	ab
EVENA-14	26.2	ab	108.4	b	45.9	110.8	ab	2.9	ab	4.3	d	−23.3	a
EVENA-15	30.5	ab	99.3	ab	46.9	112.9	ab	2.8	ab	3.3	ab	−22.8	ab
ENTAV-136	27.7	ab	100.0	ab	43.8	121	b	2.6	a	3.7	bc	−22.3	b
ENTAV-435	27.7	ab	105.2	ab	46.5	119.1	ab	3.1	b	4.3	d	−23.0	ab
RJ21	23.7	a	105.5	ab	45.7	111.7	ab	3.1	b	4.0	cd	−22.8	ab
VNQ	23.4	a	100.9	ab	43.5	107.8	ab	3.0	ab	3.7	bc	−23.5	a

Within each row, different letters indicate significantly different results at *p* < 0.05 (Duncan test).

**Table 6:** Ranking according to the measurements of whole plant water use efficiency (WUE_WP_), intrinsic water use efficiency (WUE_i_) and carbon isotope ratio in grapes (δ^13^C) for the 9 “Grenache” genotypes grown in Mallorca, Spain.

Genotype	WUE_i_ (µmol CO_2_ mol H_2_O^−1^)			WUE_WP_ (g dw L^−1^)			δ^13^C (‰)	Mean Ranking
	2020	2021	Avg.	2020	2021	Avg.	2021	
ARA-24	2	2	2	2	3	3	1	2
EVENA-11	1	2	1	1	2	2	2	1
EVENA-13	3	3	3	2	3	3	2	3
EVENA-14	2	2	2	2	1	2	1	1
EVENA-15	2	2	2	2	3	3	2	3
ENTAV-136	2	1	1	3	2	3	1	1
ENTAV-435	2	2	2	1	1	1	2	1
RJ21	2	2	2	1	1	1	2	1
VNQ	3	2	3	2	2	2	3	3

## Data Availability

The data presented in this study are available on request from the corresponding author. The data are not publicly available due to the fact that they are subject to further work.

## Supplementary Materials

The following supporting information can be downloaded at: https://www.mdpi.com/article/10.3390/plants11213008/s1. Table S1: Meteorological conditions during the experimental seasons (May–September) of 2020 and 2021 in Mallorca, Balearic Islands, Spain. Table S2: Linear regressions between stomatal conductance (g_s_) and stem water potential (Ψ_stem_) of all the genotypes across 2020 and 2021. Table S3: Linear regressions between the natural logarithm of intrinsic water use efficiency (WUE_i_) and stomatal conductance (g_s_) of all genotypes across 2020 and 2021.
