# Supplementary material for: Unraveling the Physiological Mechanisms Underlying the Intracultivar Variability of Water Use Efficiency in Vitis vinifera “Grenache”
Source: Plants (Basel). 2022 Nov 7;11(21):3008. doi: 10.3390/plants11213008 (PMC9654430; doi:10.3390/plants11213008)
Supplement: Supplementary file 1 [file plants-11-03008-s001.zip › plants-1959533-supplementary.pdf]

plants

MDPI

Table S1. Meteorological conditions during the experimental seasons (May-September) of 2020 and 2021 in Mallorca, Balearic Islands, Spain.

Year	Month	Temperature (ºC)	Relative humidity (%)	Rainfall (mm)	ET_o (mm)
2020	May	18.3 ± 0.1	69.7 ± 0.2	46.2	126.8
	June	29.2 ± 0.1	67.7 ± 0.3	228.8	150.1
	July	25.9 ± 0.1	63.6 ± 0.3	10.2	175.2
	August	26.3 ± 0.1	84.5 ± 0.2	12.4	141.8
	September	24.3 ± 0.1	74.3 ± 0.1	29.6	90.8
2021	May	20.3 ± 0.3	63.7 ± 0.2	18.8	134.6
	June	22.4 ± 0.5	66.1 ± 0.3	29.2	144.7
	July	26.5 ± 0.4	56.9 ± 0.3	3.4	153.5
	August	26.2 ± 0.3	64.2 ± 0.2	22.0	135.9
	September	22.2 ± 0.4	68.2 ± 0.3	53.6	87.4

Temperature and relative humidity are daily averages, while rainfall and reference evapotranspiration (ET_o) are monthly totals.

Table S2. Linear regressions between stomatal conductance (g_s) and stem water potential (Ψ_{stem}) of all genotypes across 2020 and 2021.

Genotype	g_s - Ψ_{stem}				
	y	a		r^2	*p*-value
ARA-24	0.5077	0.3427	ab	0.77	<0.0001
EVENA-11	0.4615	0.332	ab	0.63	<0.0001
EVENA-13	0.4802	0.3228	ab	0.79	<0.0001
EVENA-14	0.4644	0.3096	ab	0.63	<0.0001
EVENA-15	0.4707	0.329	ab	0.64	<0.0001
ENTAV-136	0.4801	0.3264	ab	0.73	<0.0001
ENTAV-435	0.5177	0.3828	a	0.64	<0.0001
RJ21	0.4658	0.2979	b	0.61	<0.0001
VNQ	0.5662	0.4019	a	0.80	<0.0001

"y" indicates the intercept, "a" the slope of the regression; "r^2" the correlation coefficients and "p-values" the significance of the regressions. Different letters indicate significant differences between regressions ($p<0.05$).

Table S3. Linear regressions between the natural logarithm of intrinsic water use efficiency (WUE_i) and stomatal conductance (g_s) of all genotypes across 2020 and 2021.

Genotype	Ln WUE_i - g_s				
	y	a		r^2	*p*-value
ARA-24	4.9964	-3.2214	b	0.82	<0.0001
EVENA-11	4.9985	-3.322	ab	0.75	<0.0001
EVENA-13	5.1129	-4.1424	a	0.88	<0.0001
EVENA-14	5.0972	-3.9286	ab	0.86	<0.0001
EVENA-15	5.0614	-3.5553	ab	0.87	<0.0001
ENTAV-136	5.0824	-3.6546	ab	0.90	<0.0001
ENTAV-435	5.0059	-3.4066	ab	0.91	<0.0001
RJ21	5.1039	-3.9491	ab	0.94	<0.0001
VNQ	5.0199	-3.3842	ab	0.88	<0.0001

"y" indicates the intercept, "a" the slope of the regression; "r^2" the correlation coefficients and "p-values" the significance of the regressions. Different letters indicate significant differences between regressions ($p<0.05$).
